# Incidence of Developmental Venous Anomalies in Patients With Multiple Sclerosis: A 3 Tesla MRI Study

**DOI:** 10.3389/fneur.2022.824347

**Published:** 2022-03-29

**Authors:** Marton Magyar, Thomas Gattringer, Christian Enzinger, Eva Hassler, Richard Partl, Michael Khalil, Gernot Reishofer, Hannes Deutschmann, Franz Fazekas

**Affiliations:** ^1^Department of Radiology (Division of Neuroradiology, Vascular and Interventional Radiology), Medical University of Graz, Graz, Austria; ^2^Department of Neurology, Medical University of Graz, Graz, Austria; ^3^Department of Radiation Oncology, Medical University of Graz, Graz, Austria

**Keywords:** multiple sclerosis, developmental venous anomaly (DVA), central nervous system, magnetic resonance imaging, clinically isolated syndrome (CIS)

## Abstract

**Objectives:**

There is evidence of involvement of the venous system in multiple sclerosis (MS). If this bears also an association with the frequency and extent of developmental venous anomalies (DVA) still has to be determined. We therefore investigated this in patients with different phenotypes of MS and in comparison, to a control population.

**Methods:**

We analyzed the contrast-enhanced T1-weighted MR scans of 431 patients (clinically isolated syndrome—CIS, *n* = 108; MS, *n* = 323) and of 162 control individuals for the presence of a DVA. We also measured the size of the DVA and draining vein and compared the DVA frequency between MS phenotypes.

**Results:**

A DVA was found in 38 (8.8 %) of patients with CIS or MS and in 11 (6.8%) controls (*p* = 0.4). DVA frequency was highest in CIS (14.8%) and lowest in progressive MS (4.0%). The mean cranio-caudal and axial extension of the DVA was significantly lower in MS patients than controls (*p* < 0.05).

**Conclusions:**

The frequency of DVA in MS patients is comparable to that in controls. Whether DVA size and appearance may change over time will have to be investigated in a longitudinal manner and with larger sample size.

## Introduction

Multiple sclerosis (MS) is the most frequent inflammatory demyelinating disease of the central nervous system with a predilection for the first clinical manifestation in the third and fourth age decades. Despite intensive research, the aetiopathogenesis of MS has not yet been fully understood but autoimmune mechanisms appear to play a central role (1). It is also well-established that MS lesions evolve predominantly around venous structures (2) which gives lesions next to or involving the corpus callosum a radiating appearance especially on midsagittal scans (3). This appearance has also received the eponym of Dawson's fingers (1).

Furthermore, there is abundant evidence of a change in the architecture of the venous system with ongoing MS (4–6). This has also led to speculations on specific characteristics of the cerebral venous system of MS patients such as a higher vulnerability of the vascular wall (6). In this context it has been suggested that there might be an increased incidence of developmental venous anomalies (DVA) in MS (7). DVA are the most common form of cerebral vascular malformations and their incidence in a large autopsy series was 2.6% (8).

Accordingly, previous MRI studies indicated a relatively low incidence of DVA in the normal population of ~1–3% (9–11). The introduction of new MRI techniques in recent years, first at 1.5T and later at 3T, has led to an increased detection rate of DVA as an incidental finding in the normal population (2.6–13.3%) (12–16). Interestingly, studies in MS patients have described a relatively variable frequency of DVA. Some publications have reported rates of 7.6–14.3% (14, 16), whereas other studies indicated even higher frequencies of 26.9% and 29.5% (15, 17). Another recent study showed that the presence of DVA did not significantly differ between the MS and control groups (18).

This discrepancy in existing data prompted us to investigate the frequency, but also anatomic location and size of DVA in patients with MS in comparison to individuals of the same age range who underwent contrast-enhanced MRI for other reasons. Presence of DVA in MS was also associated with patients' demographic data and the phenotype of the disease.

## Materials and Methods

### Patients and MR Data

For this study we analyzed the data of a cohort of patients which has been prospectively collected at the MS outpatient clinic of our primary and tertiary care university hospital over a 5-year period between 2008 and 2013. The diagnosis of MS or of a clinically isolated syndrome was based on criteria current at the time of data formulation (2010) (19–21). We also recorded patients' age, gender, disease phenotype (21) and disease severity according to the Expanded Disability Status Scale (EDSS) (22). An age between 18 and 55 years and the availability of 3 T MRI (Siemens Tim Trio, Siemens Healthineers, Erlangen, Germany) with T1 contrast-enhanced sequences served as inclusion criteria. In case of multiple MR examinations, the first examination together with the corresponding clinical and demographic data was used for analysis. We thus identified 431 patients.

One hundred and sixty-two patients without MRI pathology in the same age range who had undergone an MRI examination at 3T because of suspected meningitis or in the context of an extracranial MR angiography served for comparison as this also entails the acquisition of a T1 contrast-enhanced sequence according to our routine imaging protocols. These individuals were retrospectively identified using our electronic in-hospital database. Patients with structural lesions apart from white matter hyperintensities such as infarcts were excluded in order not to overlook vascular malformations due to brain defects. Rate of abnormal signal increase in adjacent tissue of DVA has not been addressed.

Patients with CIS or MS were investigated with an MR protocol that included T1-weighted TSE scans before and after the application of Gadolinium (0.1 mmol/ kg bodyweight) and were obtained with a slice thickness of 2 mm, repetition time (TR) of 1400 ms, echo time (TE) of 2.58 ms and field of view (FOV) of 256 mm^2^. The corresponding T1-weighted TSE scans of controls were obtained with a slice thickness of 5 mm, repetition time (TR) of 690 ms, echo time (TE) of 17 ms and field of view (FOV) 210 mm^2^.

The study was approved by the ethics committee of the Medical University of Graz, Graz, Austria.

### MR Data Evaluation

**Figure 1:**
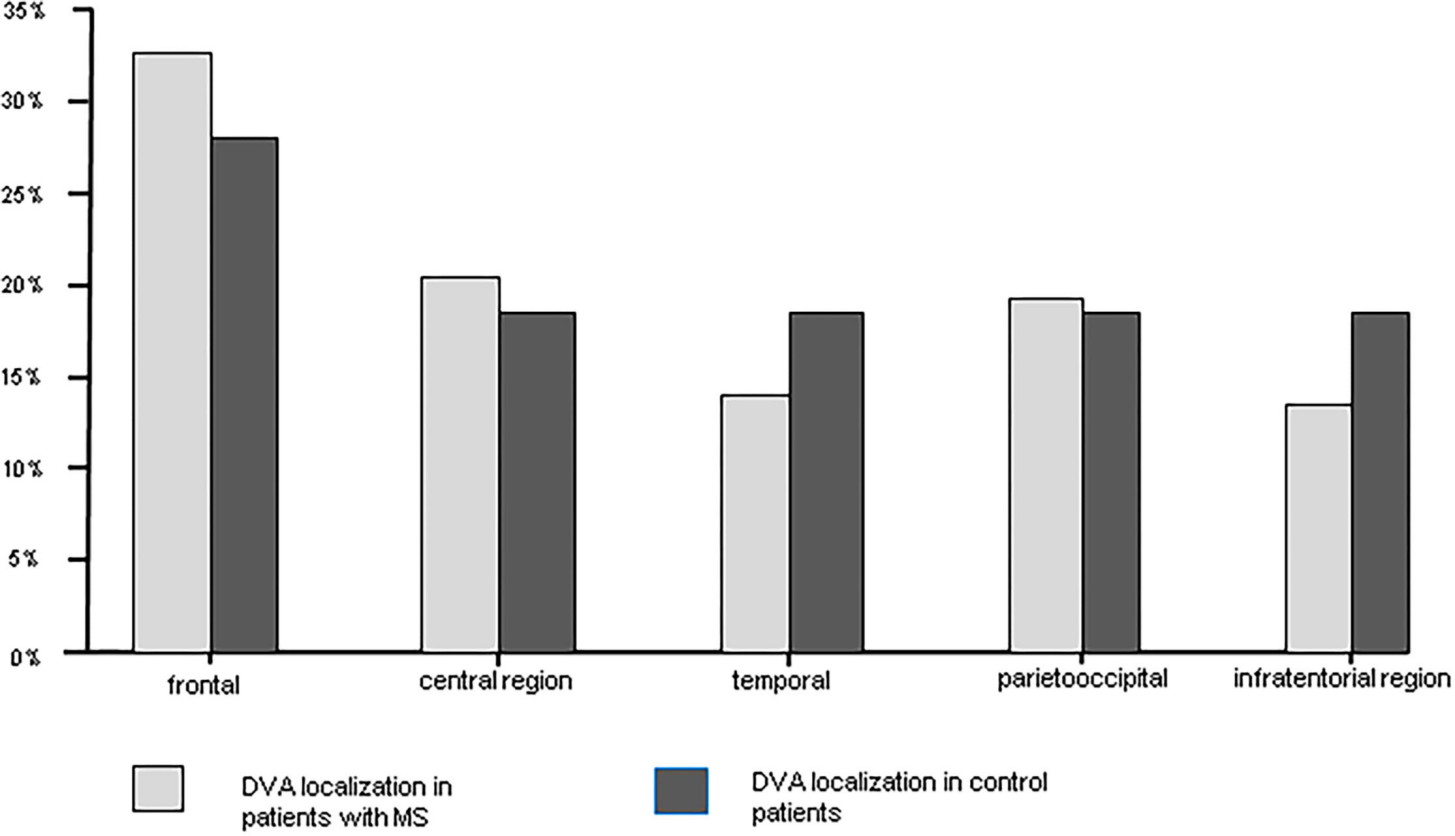
DVA localization in patients with MS and in control patients.

Image analysis was performed by a neuroradiologist with 4 years of experience who was unaware of the clinical data in question and initially reviewed the inclusion criteria for imaging as described above. Questionable cases (9 cases in total) were discussed for consensus with another neuroradiologist with more than 10 years of experience. DVA were identified on the T1 contrast enhanced scans as lesions with radial vascular structures (umbrella sign) converging toward a large collecting vessel (caput medusae) draining into either the deep or superficial venous system (Figure 1) (23). The anatomic location of DVA was defined according to the site of the collecting vein. All measurements were performed in two or three planes. Because multiple DVAs are not strictly transverse, coronal, or sagittal, expansion was more frequently evaluated in different planes because of the partial volume effect. The extent of the caput medusa was measured in the area of maximal extension of the vascular malformation. The diameter of the draining vein was measured approximately in the middle section of the vessel in mm (6), where the images were sharpest (less partial volume effect) (24, 25) and there is little conism (near the base local expansion zone with caliber irregularities and near the caput medusa many venous overlays). The length of the vessel was determined by the distance between the base and the tip of the caput medusa.

### Statistical Analysis

The statistical interpretation of the data was done with the statistics program SPSS 22.0 (SPSS Inc. Chicago, Illinois, USA). The chi-square-test according to Pearson was used to compare differences in frequency and the two-sample-*t*-test was used to compare continuous data between groups.

## Results

Demographic and clinical characteristics of patients and controls are shown in Table 1. One hundred and eight of the patients suffered from CIS and 332 had a diagnosis of MS. MS was of a relapsing remitting (RRMS) type in 224 (52%) and secondary progressive (SPMS) in 43 (10%). Two (0.5%) patients had primary progressive MS and the exact disease course could not be retrieved in 54 (12.5%). Mean age was significantly higher in the controls than patients (39.64 yrs in controls vs. 36.7 yrs in patients, *p* = 0.003) and there was a significantly higher proportion of females in the patient group (49.4% in controls vs. 66.1% in patients, *p* < 0.001).

**Table 1:** Demographic and clinical data of patients and controls.

	**Patients**,	**Controls**,	***p***
	***n* = 431**	***n*= 162**	
Females, *n* (%)	285 (66.1)	80 (49.4)	<0.001^a^
Age in years, mean (SD)	36.7 (10.5)	39.64 (10.9)	0.003^b^
Clinically isolated syndrome, *n* (%)	108 (25.1)		
Multiple sclerosis, *n* (%)	323 (74.9)		
Disease modifying treatment	265 (61.5)		
EDSS, median (interquartile range)	1.5 (0.0–2.6)		

a *Chi-square-test*.

b *T-Test*.

**Figure 2:**
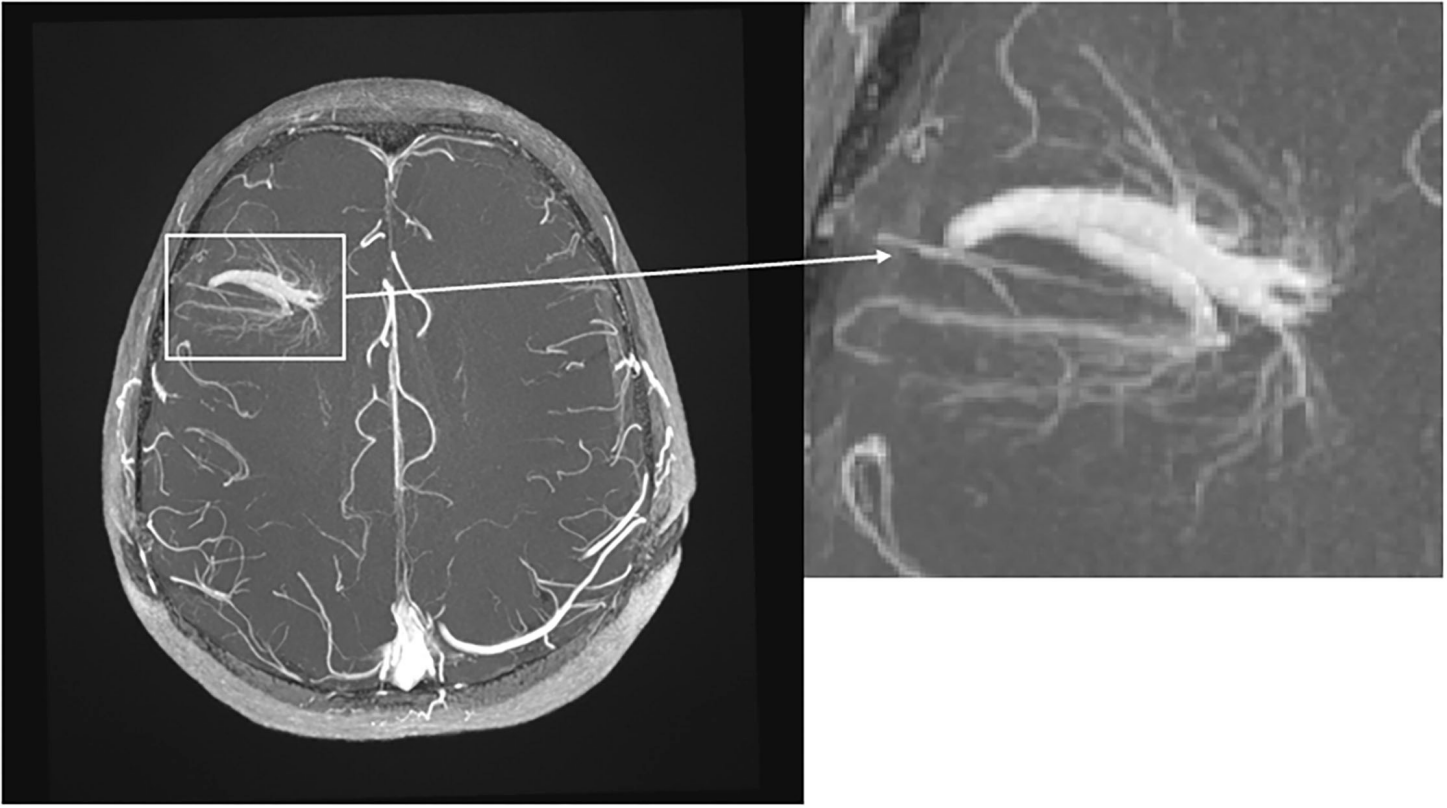
Transverse T1-weighted TOF MRI with intravenous contrast. Note the big DVA with two collecting veins in the right frontal lobe with “umbrella sign.”

**Figure 3:**
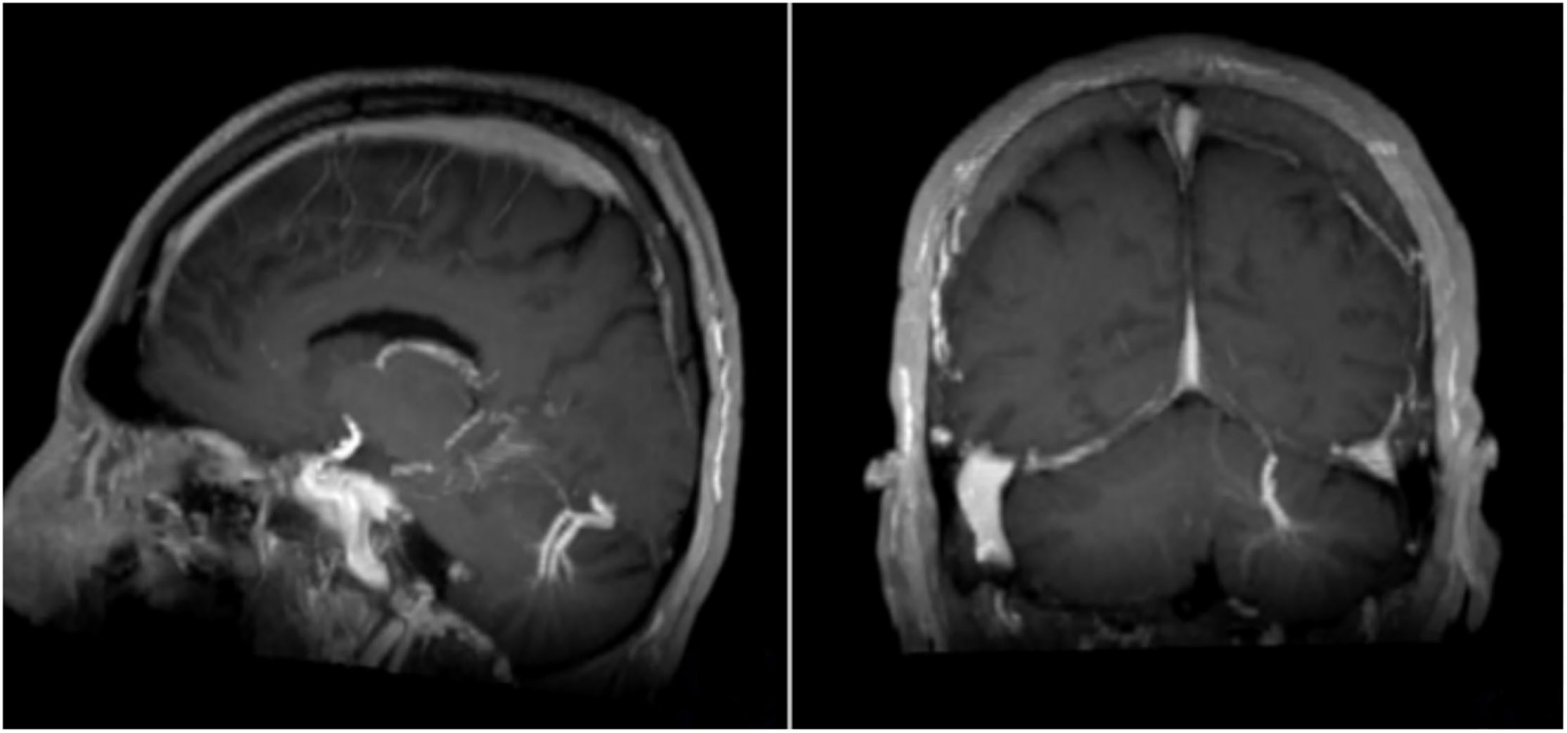
Sagittal and coronar T1-weighted MRI with intravenous contrast medium. Note the big DVA with two collecting veins cerebellar left.

Overall CIS and MS patients showed a DVA frequency of 8.8% which was not significantly higher than that in the control group with a frequency of 6.8% (*p* = 0.424) (Table 2). Using Fisher's exact test in SPSS, there was no statistically significant variance in the frequency of DVAs according to clinical phenotype. The highest frequency of DVA was seen in patients with CIS (14.8%, *p* = 0.990) and the lowest in patients with SPMS (4.0%, *p* = 0.461) while RRMS patients had an intermediate DVA frequency of 7.5% (*p* = 0.687). Regarding anatomic distribution, DVA were located most often frontally and there was no difference between patients and controls (Figure 1). Multiple DVA (two) were seen in three CIS/MS patients and in none of the control individuals (Figures 2, 3).

**Table 2:** Frequency of DVA in patients and controls.

	**Patients with/**	**Controls with/**	***p***
	**without DVA (%)**	**without DVA (%)**	
All	38/393 (8.8)	11/151 (6.8)	0.424^a^
Clinically isolated syndrome	16/92 (14.8)		0.990^b^
Relapsing remitting MS	17/207 (7.5)		0.687^b^
Secondary progressive MS	2/41 (4.0)		0.461^b^
Primary progressive MS	0/2 (0.0)		0.869^b^
Unknown phenotype	3/51 (5.5)		0.519^b^

*Subgroup frequencies were calculated individually compared with controls using a Fisher's exact test*.

a *Chi-square test*.

b *Fisher's exact test*.

Measurement results on the extent of DVA are listed in Table 3. There was no difference in the range of the maximal axial and cranio-caudal extension of DVA between groups, but the mean extension was significantly greater in controls than patients although this difference was small in absolute terms. The range in the diameter of the draining veins and their mean length and width were comparable between both groups.

**Table 3:** Extension of DVA's in patients and controls.

	**Patients**,	**Controls**,	***p***
	***N* = 38**	***N* = 11**	
Length (mm)
Range	10–44	14–40	
Mean (SD)	22.6 (8.9)	29.3 (8.4)	0.033^x^
Width (mm)
Range	1–21	5–28	
Mean (SD)	11.1 (5.6)	15.6 (6.8)	0.027^x^
Diameter of draining vein (mm)
Range	1–3	1–3	
Mean (SD)	1.7 (0.7)	1.7 (0.8)	NS^x^

*mm, millimeter*.

x *T-Test*.

## Discussion

Early studies have indicated a relatively low incidence of DVA in the normal population of around 1–3% (9–11). The implementation of novel MRI techniques in recent years has led to an increased detection rate of DVA as an incidental finding in the normal population (2.6–13.3%) (12, 13, 15). In studies on MS patients, higher DVA rates of (7.6–14.3%) have been described (14, 16). This raised the speculation that DVA might be found at an increased rate in MS in line with other data suggesting venous involvement in this disease (4). However, when comparing a relatively large group of patients with CIS suggestive of MS or definite MS to patients in whom T1-contrast enhanced scans had been obtained for other reasons, we found no significant difference in the frequency of DVA between both groups.

A higher proportion of CIS / MS patients than controls showing DVA might also have been caused by the lower slice thickness of 2 mm that was used when scanning patients compared to the regular 5 mm slice thickness used for controls although this should not have had a major impact given the generally larger size of DVA.

Interestingly, the rate of DVA declined with progressing disease, i.e., was highest in patients with CIS and lowest in patients with a progressive disease course. While these differences did not reach statistical significance and may have been just a chance finding it cannot be excluded that at least small DVA tend to disappear with progressing MS in parallel to the rarefaction of the venous system that has been suggested to occur in the course of the disease (5, 6). The notion of a lower maximal extension of the DVA in our patients compared to controls could go along with this assumption.

A similarly designed study by Sasani et al. obtained comparable results regarding the frequency of DVA in both patients and controls (12.2% with and 7.6% without MS) (14). It should be noted that in this study both groups were examined on a 1.5 T scanner and in both groups 85.5% of the patients were women (as opposed to 66% in our study population). Moreover, there is agreement with another study with a smaller MS group, where DVAs were more likely (not statistically significant) to be detected in CIS. Remarkably in this study is the much higher number of DVA in the MS group (26.9 vs. 8.8% in our study) (17). Similarly, high numbers are found in another study by Halicioglu et al. with about 105 subjects in both groups and with a probability on DVA of 29.5% in MS and 13.3% in the control group (vascular headache without MS like lesions) (15).

It is expected that 3D sequences will detect smaller DVA than 2D sequences due to gapless/isotropic imaging and the possibility of reformatting. On older MRI scanners, routine imaging with 3D T1-w TSE was not yet possible because of the substantial time requirements. For this reason, post-contrast 3D GRE sequences (MPRAGE) were used, which mostly have the disadvantage of lower contrast. Consequently, the detection of contrast-absorbing structures, such as metastases, and active MS lesions was more difficult. For this reason, at the time of data collection, MS patients in our clinic were routinely screened with 2D T1-w SE. In addition, the frequently occurring flow or pulsation artifacts make a reliable detection of lesions in the area of large intracranial vascular structures difficult. All these reasons may be responsible for the noted differences in the frequency of DVA in different studies. A better detectability of DVA can be expected from 3D T1-w TSE and from 3D FLAIR on currently available modern MRI equipment with significant reduction of artifacts and reasonable contrast after gadolinium administration (18, 26). A corresponding study at 7T and with follow-up would be interesting to see how many of these smallest DVA are present and whether they really disappear during the course of the disease.

Our group also neglected the fusion of the evaluation of FLAIR and post contrast T1 images, so unfortunately their evaluation was not performed. Moreover, at the time of data collection, T2^*^ was performed routinely instead of SWI.

However, we acknowledge that high-resolution 3D susceptibility-weighted imaging (SWI) might have a higher sensitivity for DVA detection. Interestingly, studies using SWI in this context are sparse (18, 27, 28). Cho et al. (12) compared the detection rate of T1 post-contrast images (gold standard) with SWI, T2, and FLAIR in a total of 28 patients and found a high sensitivity of more than 85.7% in detecting DVA with SWI and a much lower sensitivity with T2 and FLAIR (35.7%). Specificity was above 90% with all three methods (92.9, 92.9, and 96.4 with FLAIR). Of interest is the overall very low detection rate of 28 patients with DVA in their group of 1,068 MRI examinations evaluated (2.6%).

Another limitation of our study is the lack of evaluation of FLAIR signaling abnormalities associated with DVA in patients with MS (28–31).

In this context, Rogers et al. investigated whether there was a significant prevalence of MS plaques associated with DVA (29). Their group, using multivariate logistic regression, accounting for several possible confounding variables, found that DVA surrounding white matter lesions were 6.7 times more common in MS patients than in the control group (47.3 vs. 13.5%, *p* < 0.001), suggesting that instead of global cerebrospinal venous insufficiency, there could be rather a local impairment of venous outflow in the DVA area. Development of MS plaques around DVA could be a result of blood-brain barrier breakdown and local lymphocytic infiltration, however this has to be explored further.

Although we analyzed relatively large groups of patients with CIS/MS and controls, the absolute number of individuals with DVA was still relatively small and does not allow for solid within group comparisons, which needs to be acknowledged as a significant limitation of our study. Furthermore, it would need a longitudinal study design to substantiate claims of a change in DVA extension during the evolution of MS. Controls in our study were also older and had a lower proportion of females than the MS group, but these factors have not yet been associated with DVA frequency and it is therefore unlikely that they would have influenced our result to a greater extent. Irrespective of these limitations our study provides strong evidence that patients with MS do not suffer from cerebral venous anomalies in the form of a DVA more frequently than the general population.

## Data Availability Statement

The original contributions presented in the study are included in the article/supplementary material, further inquiries can be directed to the corresponding author/s.

## Ethics Statement

The studies involving human participants were reviewed and approved by Ethikkommission der Medizinischen Universität Graz. Written informed consent for participation was not required for this study in accordance with the national legislation and the institutional requirements.

## Author Contributions

MM, TG, CE, EH, RP, and FF contributed to conception and design of the study. MK organized the database. GR, CE, and MM performed the statistical analysis. MM wrote the first draft of the manuscript. MM, TG, CE, and FF wrote sections of the manuscript. All authors contributed to manuscript revision, read, and approved the submitted version.

## Conflict of Interest

The authors declare that the research was conducted in the absence of any commercial or financial relationships that could be construed as a potential conflict of interest.

## Publisher's Note

All claims expressed in this article are solely those of the authors and do not necessarily represent those of their affiliated organizations, or those of the publisher, the editors and the reviewers. Any product that may be evaluated in this article, or claim that may be made by its manufacturer, is not guaranteed or endorsed by the publisher.
